# Editorial: Infant food and intestinal immunity

**DOI:** 10.3389/fnut.2022.1089890

**Published:** 2022-11-25

**Authors:** Yingwang Ye, Stephen Forsythe, Zhenbo Xu

**Affiliations:** ^1^School of Food and Biological Engineering, Hefei University of Technology, Hefei, China; ^2^Independent Researcher, Nottingham, United Kingdom; ^3^Department of Laboratory Medicine, The Second Affiliated Hospital of Shantou University Medical College, Shantou, China

**Keywords:** infant food, intestinal health, intestinal immunity, intestinal microflora, intestinal inflammation

This Research Topic, led by Dr. Yingwang Ye, Dr. Stephen Forsythe, and Dr. Zhenbo Xu, has contained a total of six excellent manuscripts. As the background of this Research Topic was concerned, infant and young child food have received special attention from the government and society as a type of specialized food. In recent years, there have been numerous outbreaks of infections and poisons in infant food. As a result, quality control and government oversight are becoming more stringent over the world (1). Infant food is a type of nourishment that has a finely balanced composition that aims to mimic breast milk, the gold standard, as nearly as possible. The baby food industry nowadays offers a wide range of products trying to fulfill the changing needs of newborns and young children in their early stages of life (2). In addition, infant food serves as food for a group of our population that is more vulnerable to pathogens and toxins. As concluded, the research about infant food and intestinal immunity is of importance for infant health and development (3–5). As a consequence, this Research Topic aims to contribute to providing the knowledge about the relationship between infant food and intestinal immunity by presenting current and novel research being to obtain information about intestinal health, intestinal immunity, intestinal microflora, intestinal inflammation, which will guide consumption of infant food and its production. As article type was concerned, this Research Topic welcomes submissions of Original Research, Review and Mini-Review articles focus on (but not limited to) infant formula and intestinal health, infant nutrition and microflora, intestinal inflammation, as well as toxins in infant food and intestinal immunity. Key words of this Research Topic include infant food, intestinal health, intestinal immunity, intestinal microflora, and intestinal inflammation. As stated above, a total of six manuscripts are accepted in this Research Topic, with three published in Frontiers in Nutrition and three published in Frontiers in Immunology.

Since gut microbiota has been well-documented to be important for intestinal health, a large proportion of the published manuscripts in this Research Topic are relevant to gut microbiota.

The first published manuscript in this Research Topic entitled “*The gut microbiome of preterm infants treated with aminophylline is closely related to the occurrence of feeding intolerance and the weight gain*” by Shen et al., has studied the gut microbiome of preterm infants treated with aminophylline. *Via* a cohort analysis on 118 preterm infants, the authors have found out gut microbiome in preterm infants treated with aminophylline was characterized by a decrease in *Streptococcus* and *Rothia* and increase in *Staphylococcus*, whereas *Rothia* positively correlated with the occurrence of feeding intolerance and *Streptococcus* but not Bifidobacter likely participated in the weight gain of preterm infants in early life. The second published manuscript, entitled “*Intrahepatic cholestasis of pregnancy increases inflammatory susceptibility in neonatal offspring by modulating gut microbiota*” by Lin et al., had investigated the effect of maternal cholestasis on neonatal offspring metabolism and immune function. In comparing neonates with Intrahepatic cholestasis of pregnancy (ICP) mothers (*n* = 71) and healthy mothers (*n* = 63), the authors had indicated that cholestatic pregnancy may increase the susceptibility of the offspring to inflammation by altering bile acid metabolism and gut microbiota at an early stage, and also supplementation with *Lactobacillus rhamnosus* LRX01 inhibits FXR expression in the ileum and thus may improve intestinal immunity in ICP offspring. Such finding has provided important knowledge on how ICP shapes the offspring's immunity and predisposition to inflammatory disorders at an early stage. The third published manuscript, entitled “*Lactobacillus casei SYF-08 protects against Pb-induced injury in young mice by regulating bile acid metabolism and increasing Pb excretion*” by Chen et al., had aimed at identifying a non-toxic treatment on Pb poisoning by studying the therapeutic effect of an infant feces-derived probiotic strain, *Lactobacillus casei* SYF-08 (SYF-08), on Pb poisoning in young mice. The authors had eventually provided valuable insights into the therapeutic role of SYF-08 in Pb poisoning and also suggested that its administration can significantly alleviate the Pb-induced damage. The fourth published manuscript in this Research Topic, entitled “*Dietary supplementation of chitosan oligosaccharide–Clostridium butyricum synbiotic relieved early-weaned stress by improving intestinal health on pigeon squabs (Columba livia)*” by Wen et al., was a follow-up study from previous finding whereas the authors had discovered early weaning causes harm to growth performance, intestinal morphology, activity of digestive enzymes, and antioxidant status in pigeon squabs (*Columba livia*). Consequently, in this study, the authors further explored whether dietary supplementation with COS-C. butyricum synbiotic could relieve early-weaned stress by evaluating its effects on growth performance and intestinal health in pigeon squabs. As found, dietary supplementation with synbiotic (150 mg/kg COS + 300–400 mg/kg *C. butyricum*) could relieve early-weaned stress by maintaining intestinal health in pigeon squabs. The fifth published manuscript in this Research Topic is a retrospective study, entitled “*Effect of different feeding methods and gut microbiota on premature infants and clinical outcomes*” by Liu et al., had investigated the effects of two routine feeding methods, breastfeeding and formula milk, on premature infants. *Via* statistical analysis on premature infants admitted to the neonatal intensive care unit between 2017 and 2018, the authors had obtained no significant differences between the two feeding methods, neither had significant effects on clinical indicators in premature infants. Gut microbiota has been well-studied in influencing many clinical indicators, this study suggests an important role of gut microbiota plays in the care of premature infants and further provides a basis for promoting the healthy development of this patient population. The sixth manuscript published in this Research Topic is a highlight study, entitled “*Bacteroides fragilis ameliorates Cronobacter malonaticus lipopolysaccharide-induced pathological injury through modulation of the intestinal microbiota*” by Ling et al. As well-studied, *Cronobacter* has been considered to be an important pathogen which is responsible for meningitis and necrotizing enterocolitis (NEC) in newborns, and lipopolysaccharide (LPS) has been identified to commonly facilitate bacterial translocation along with inflammatory responses as an endotoxin. Based on the unclear pathogenicity of *Cronobacter* LPS and the potential strategies to alleviate the toxicity, the authors had stimulated inflammatory responses in Sprague–Dawley young rats by intraperitoneal injection of *Cronobacter malonaticus* LPS, and fed with *Bacteroides fragilis* NCTC9343 continuously fed through gavage for 5 days before or after injection, to further evaluate the intervention effect of *B. fragilis*. As found, *C. malonaticus* LPS exacerbated intestinal infection by altering gut microbe profile, tight junction protein expression, and releasing inflammatory factors in a time- and dose-dependent manner, in which pathological injuries could be diminished by *B. fragilis* treatment. *Shigella, Peptoclostridium*, and *Sutterella* were found to be positively related to *C. malonaticus* LPS infection, with *Prevotella* negatively correlated. Collectively, the authors had concluded the intestinal microbiota is an important target for the prevention and treatment of pathogenic injuries induced by *C. malonaticus* LPS, which has provided a potential strategy to alleviate the toxicity.

As concluded, the above manuscripts published in this Research Topic, had provided comprehensive knowledge and insight in understanding the relationship between infant food and intestinal immunity *via* studies on intestinal health, intestinal immunity, intestinal microflora, intestinal inflammation, which will further guide consumption of infant food and its production. As seen, gut microbiota modulation, probiotic and its application, as well as microbial pathogenicity based novel strategy for toxicity alleviation, are research hotspots in infant food and intestinal immunity. Up-to-date, this Research Topic has achieved 6,681 total views and 1,424 downloads.

## Author contributions

All authors listed have made a substantial, direct, and intellectual contribution to the work and approved it for publication.

## Conflict of interest

The authors declare that the research was conducted in the absence of any commercial or financial relationships that could be construed as a potential conflict of interest.

## Publisher's note

All claims expressed in this article are solely those of the authors and do not necessarily represent those of their affiliated organizations, or those of the publisher, the editors and the reviewers. Any product that may be evaluated in this article, or claim that may be made by its manufacturer, is not guaranteed or endorsed by the publisher.
